# A new species of the *Clubionacorticalis*-group (Araneae, Clubionidae) from Jiugong Mountains, Hubei Province, central China

**DOI:** 10.3897/BDJ.10.e94735

**Published:** 2022-10-24

**Authors:** Yang Zhong, Xusheng Gong, Hao Yu

**Affiliations:** 1 Administrative Commission of Jiugongshan National Nature Reserve of Hubei Xianning, Xianning, China Administrative Commission of Jiugongshan National Nature Reserve of Hubei Xianning Xianning China; 2 School of Nuclear Technology and Chemistry & Biology, Hubei University of Science and Technology, Xianning, China School of Nuclear Technology and Chemistry & Biology, Hubei University of Science and Technology Xianning China; 3 School of Life Sciences, Guizhou Normal University, Guiyang, China School of Life Sciences, Guizhou Normal University Guiyang China

**Keywords:** Sac spiders, morphology, DNA barcoding, diagnosis, taxonomy

## Abstract

**Background:**

The *corticalis* group is one of most diverse species-group in genus *Clubiona* Latreille, 1804. Currently, a total of 81 *corticalis* group species are known worldwide, amongst them 67 were recorded from China. However, the diversity of this group in China is still insufficiently known.

**New information:**

*Clubionaxianning* **sp. nov.** is described as a new species of the *C.corticalis* species-group collected from Hubei Province, China.

## Introduction

*Clubiona* Latreille, 1804 currently contains 518 catalogued species that are found worldwide, except for the Polar Regions and South America (World Spider Catalog 2022), is the most diverse genus in the family Clubionidae and one of the most diverse genera in the order Araneae (Zhang and Yu 2020, Zhang et al. 2021, World Spider Catalog 2022). One of the generic species-groups, the *C.corticalis*-group, exhibits high species diversity and currently contains 81 species (Zhang et al. 2021). Up to now, there are 67 described *corticalis* group species distributed in China, making it one of the most diverse clubionid groups in China (Zhang et al. 2021). However, the diversity of this group in China is still insufficiently known and several new species have been described in the last few years (Zhang et al. 2018, Yu and Li 2019a, Yu and Li 2019b, Zhang and Yu 2020, Zhang et al. 2021).

**Figure 1.**
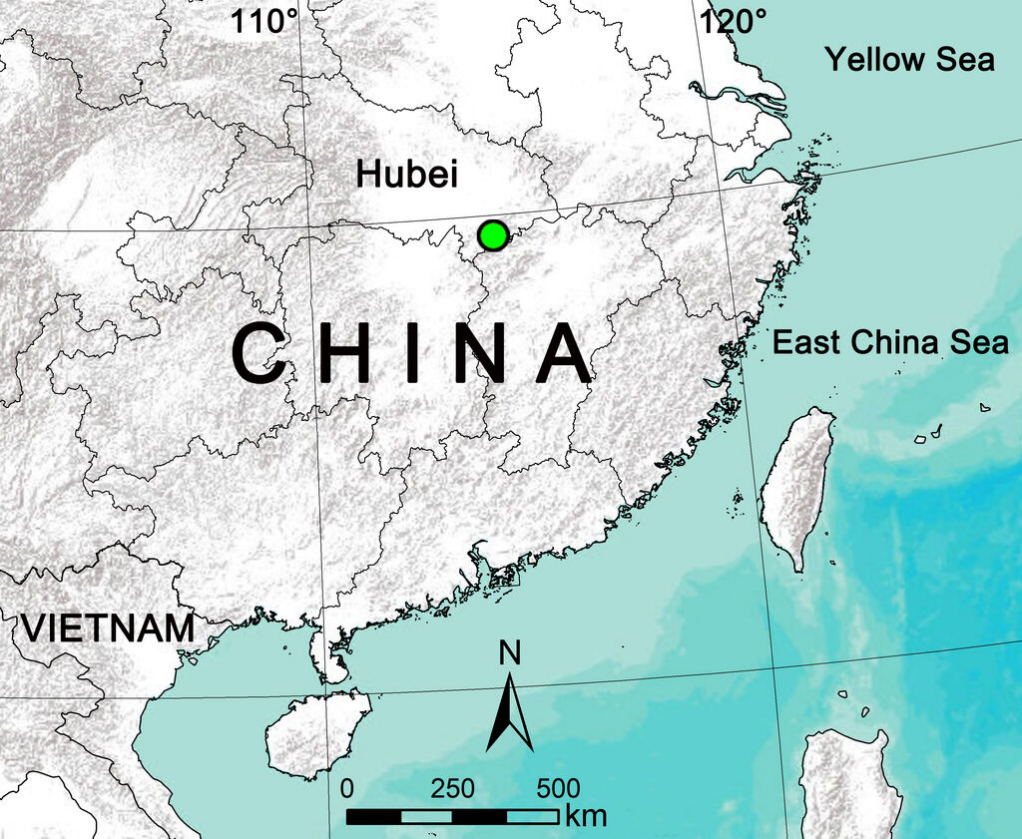
Distribution record of *Clubionaxianning* **sp. nov.** (green circle).

While examining spiders collected from Jiugong Mountains, Hubei Province, China (Fig. 1), we found pairs of *Clubiona* specimens that belong to an undescribed species of the *corticalis*-group similar to *C.caohai* Zhang & Yu, 2020 and *C.altissimus* Hu, 2001. The goal of this paper is to provide a detailed description and diagnosis of the new species. The DNA barcodes of the new species were obtained for gender matching and future use in molecular studies.

## Materials and methods

Specimens in this study were collected by hand collecting from leaf-litter in Mt. Jiugong, Hubei. Spiders were fixed and preserved in 95% ethanol. Specimens were examined with an Olympus SZX7 stereomicroscope; details were studied with an Olympus CX41 compound microscope. Female epigyne and male palp were examined and illustrated after being dissected. The epigyne was removed and cleared in warm lactic acid before illustration. The vulva was also imaged after being embedded in Arabic gum. Photos were made with a Cannon EOS70D digital camera mounted on an Olympus CX41 compound microscope. The digital images were taken and assembled using Helifocus 6.80 software package. The distribution map was generated with Arcgis 10.5 (Environmental Systems Research Institute, Inc.).

A DNA barcode was also obtained for species matching. A partial fragment of the mitochondrial cytochrome oxidase subunit I (CO1) gene was amplified and sequenced for two specimens, using the primers LCOI1490 (5’-GGTCAACAAATCATAAAGATATTG-3’) and HCOI2198 (5’-TAAACTTCAGGGTGACCAAAAAAT-3’) (Folmer et al. 1994). For additional information on extraction, amplification and sequencing procedures, see Wheeler et al. (2016). DNA sequences were checked and edited with BioEdit 7.2.2 (Hall 1999), sequences being trimmed to 653 bp. Sequence alignment was completed using CLUSTAL W (Thompson et al. 1994). Genetic distances were computed with MEGA 5 (Tamura et al. 2011). All sequences were confirmed using BLAST and are deposited in GenBank. The codes and GenBank accession numbers of voucher specimens are provided as follows: YHCLU0272, ♂, GenBank OP675437; YHCLU0273, ♀, GenBank OP675436.

All measurements were obtained using an Olympus SZX7 stereomicroscope and given in millimetres. Eye diameters are taken at the widest point. The total body length does not include chelicerae or spinnerets length. Leg lengths are given as total length (femur, patella, tibia + metatarsus, tarsus). Most of the terminologies used in text and figure legends follows Zhang et al. (2021), while a few others followed Zhang et al. (2018) and Zhang and Yu (2020).

All specimens are deposited Museum of Guizhou Normal University, Guiyang, Guizhou, China.

## Taxon treatments

### Clubiona xianning

Zhong & Yu sp. n.

B0CC6F23-8DFB-5B4A-BAD0-E8AC5114D687

8DFF1CC4-1C1A-46A6-8300-FF36FFFA2644

#### Materials

- **Type status:** Holotype. **Occurrence:** recordedBy: Yang Zhong, Xusheng Gong, Qianle Lu; individualID: YHCLU0272; individualCount: 1; sex: male; lifeStage: adult; behavior: foraging; preparations: whole animal (ETOH); associatedSequences: GenBank: OP675437; occurrenceID: 1312422A-3320-5AE6-9BA9-5DCB3013B14F; **Taxon:** order: Araneae; family: Clubionidae; genus: Clubiona; specificEpithet: *xianning*; scientificNameAuthorship: Zhong & Yu; **Location:** continent: Asian; country: China; countryCode: CHN; stateProvince: Hubei; county: Tongshan; municipality: Xianning; locality: Jiugongshan Nature Reserve; decimalLatitude: 29.39; decimalLongitude: 114.65; **Identification:** identifiedBy: Hao Yu; dateIdentified: 2022-05; **Event:** samplingProtocol: by hand; samplingEffort: 10 km by foot; year: 2020; month: 7; day: 4; **Record Level:** basisOfRecord: PreservedSpecimen
- **Type status:** Holotype. **Occurrence:** recordedBy: Yang Zhong, Xusheng Gong, Qianle Lu; individualID: YHCLU0273; individualCount: 1; sex: female; lifeStage: adult; behavior: foraging; preparations: whole animal (ETOH); associatedSequences: GenBank: OP675436; occurrenceID: 085B9104-F139-5EC1-9913-5A5DB9C7C9C6; **Taxon:** order: Araneae; family: Clubionidae; genus: Clubiona; specificEpithet: *xianning*; scientificNameAuthorship: Zhong & Yu; **Location:** continent: Asian; country: China; countryCode: CHN; stateProvince: Hubei; county: Tongshan; municipality: Xianning; locality: Jiugongshan Nature Reserve; decimalLatitude: 29.39; decimalLongitude: 114.65; **Identification:** identifiedBy: Hao Yu; dateIdentified: 2022-05; **Event:** samplingProtocol: by hand; samplingEffort: 10 km by foot; year: 2020; month: 7; day: 4; **Record Level:** basisOfRecord: PreservedSpecimen

#### Description

**Male** (Fig. 4E and F). Total length 5.31; carapace 2.27 long, 1.73 wide; abdomen 3.04 long, 1.31 wide.

**Figure 2.**
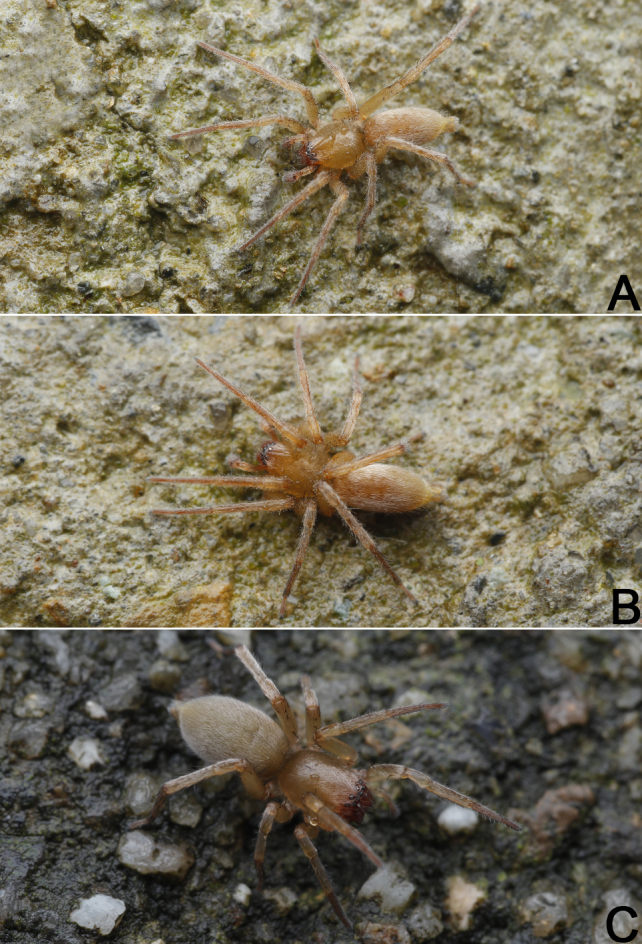
*Clubionaxianning* **sp. nov.**, male holotype (**A, B**) and female paratype (**C**), live specimens. Photographs by Qianle Lu (Shenzhen, Guangdong).

Colour of the living holotype male was uniformly brown (Fig. 2A and B). Carapace (Fig. 4E and F) elongate, oval, light brown in alcohol, uniformly coloured, without pattern, fovea red; pars cephalica distinctly narrowed, cervical groove radial groove indistinct; tegument smooth, with erect, thin, dark setae on front ridge. Eyes: in dorsal view, anterior eye row (AER) slightly recurved, posterior eye row (PER) almost straight, PER wider than AER. Eye sizes and interdistances: anterior median eyes (AME) 0.13, anterior lateral eyes (ALE) 0.12, posterior median eyes (PME) 0.11, posterior lateral eyes (PLE) 0.09, distance between AMEs (AME–AME) 0.07, distance between AME and ALE (AME–ALE) 0.03, distance between PMEs (PME–PME) 0.19, distance between PME and PLE (PME–PLE) 0.09. Length of median ocular quadrangle (MOQL) 0.33, MOQ anterior width (MOQA) 0.29, MOQ posterior width (MOQP) 0.54. Chelicerae robust, light orange, with red fangs, with four promarginal and two retromarginal teeth. Sternum nearly shield-shaped, yellowish-white, 1.21 long, 0.86 wide. Labium and endites coloured as carapace.

Abdomen (Fig. 4E and F) oval and light brown, dorsally with a wide and more or less oblong scutum extending ca. 2/3 of abdomen length, with two pairs of inconspicuous muscle depressions on either side; venter white with no distinct pattern; spinnerets yellowish-white.

Legs uniformly yellowish-white in ethanol (Fig. 4E and F). Leg length: I 5.85 (1.73, 2.32, 1.24, 0.57), II 6.29 (1.79, 2.44, 1.33, 0.73), III 5.27 (1.69, 1.60, 1.58, 0.40), IV 7.56 (2.19, 2.54, 2.31, 0.52).

**Figure 3.**
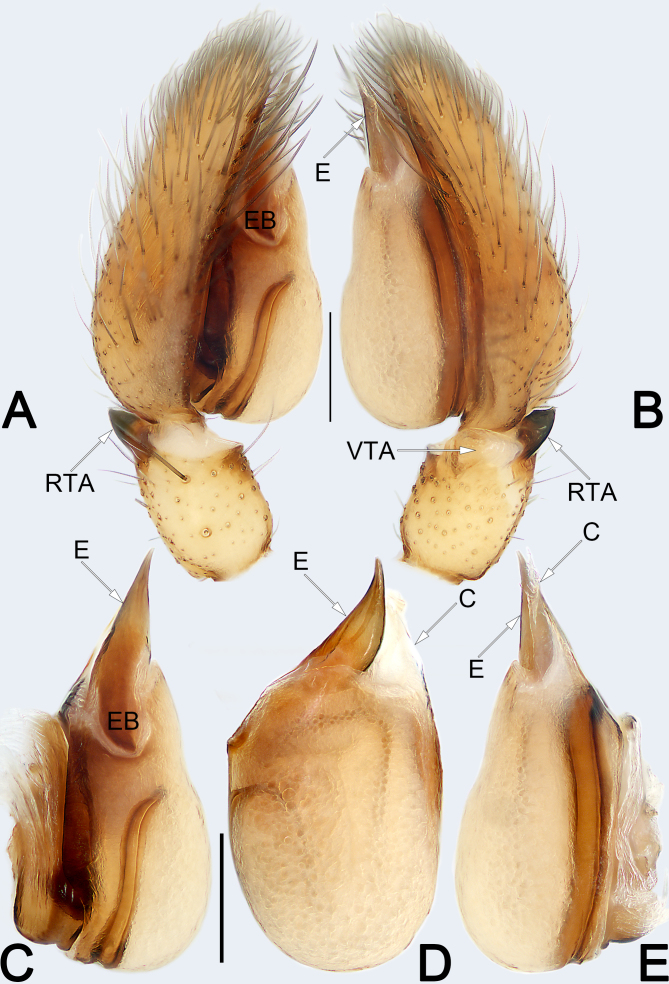
Male left palp of the holotype of *Clubionaxianning* **sp. nov. A** Prolateral view; **B** Rretrolateral view; **C** Bulb, prolateral view; **D** Bulb, ventral view; **E** Bulb, retrolateral view. Abbreviations: C = conductor; E = embolus; EB = embolic base; RTA = retrolateral tibial apophysis; VTA = ventral tibial apophysis. Scale bars: 0.2 mm (equal for **A** and **B**, equal for **C–E**).

Palp (Fig. 3A–E). Femur and patella unmodified. Tibia relatively short, about 2/5 of cymbium length, with two apophyses: a retrolateral one (RTA) that is heavily sclerotised, ca. 1/2 of palpal tibia length, more or less blade-shaped; a partly membranous, laminar apophysis (VTA), ca. 1/3 of palpal tibia length. Bulb nearly pyriform, slightly excavated on prolatero-apical side to accommodate embolus; tegulum oval and slightly expanded, ca. 1.45 longer than wide, sperm duct indistinct in venter view; subtegulum (ST) large, located prolaterally. Embolus (E) wide and heavily sclerotised, about 2/3 of the tegulum length, dagger-shaped, gradually tapering towards its apex, its tip sharp, slightly curved and extending to apex of cymbium. Conductor long and membranous, irregular-shaped in venter view and triangular in retrolateral view, its tip extending above apex of embolus.

**Figure 4.**
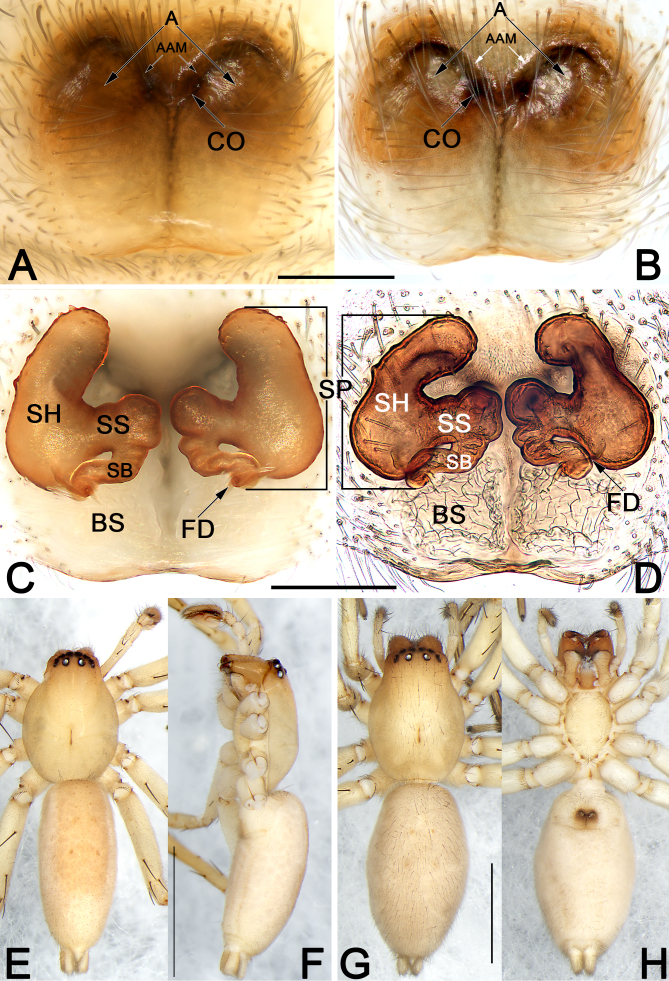
*Clubionaxianning* **sp. nov.**, female paratype and male holotype. **A** Intact epigyne, ventral view; **B** Cleared epigyne, ventral view; **C** Cleared vulva, dorsal view; **D** Vulva, cleared and embedded in Arabic gum, dorsal view; **E** Male habitus, dorsal view; **F** Male habitus, lateral view; **G** Female habitus, dorsal view; **H** Female habitus, ventral view. Abbreviations: A = atrium; AAM = atrial anterior margin; BS = bursa; CO = copulatory opening; FD = fertilisation duct; SB = spermathecal base; SH = spermathecal head; SP = spermatheca; SS = spermathecal stalk. Scale bars: 0.2 mm (equal for **A** and **B**, equal for **C** and **D**); 2 mm (equal for **E** and **F**, equal for **G** and **H**).

**Female** (Fig. 4G and H). Total length 6.15; carapace 2.64 long, 1.98 wide; abdomen 3.51 long, 1.90 wide. Eye sizes and interdistances: AME 0.16, ALE 0.15, PME 0.14, PLE 0.13; AME–AME 0.07, AME–ALE 0.04, PME–PME 0.22, PME–PLE 0.04. MOQL 0.40, MOQA 0.36, MOQP 0.53. Sternum 1.39 long, 0.96 wide. Measurements of legs: I 5.48 (1.69, 2.12, 1.05, 0.62), II 5.93 (1.90, 2.28, 1.09, 0.66), III 5.29 (1.82, 1.90, 1.14, 0.44), IV 8.07 (2.52, 2.64, 2.32, 0.59). General characters as in female, but slightly larger in size and lighter in colour.

Epigyne (Fig. 4A–D). Epigynal plate slightly wider than long, spermathecae and bursae are indistinctly visible through epigynal plate in ventral view. Atrium (A) large, represented by two symmetrical, spherical, shallow depressions; atrial anterior margin (AAM) distinctly delimited, M-shaped, posterior and lateral anterior margins not rebordered. Two copulatory openings (CO) indistinct, located at medial portion of atrial anterior margin. Spermathecae (SP) with 3 parts: spermathecal head (SH) finger-like and large, ca. 2.7x longer than diameter, the two spermathecal heads separated by 1.58 diameters; spermathecal stalk tubular, running horizontally; (SS) spermathecal base (SB) tubular and convoluted, distinctly thinner than spermathecal head and spermathecal stalk. Fertilisation ducts (FD) short and curved, acicular, located at distal end of spermathecal base.

##### DNAbarcode:

5'TTCTGGTCAGCTATAGTTGGTACAGCTATAAGAGTTATAATTCGTATAGAATTAGGTCAATCTGGAGCTTTTTTAGGTGATGATCATTTGTATAATGTAGTAGTTACTGCTCATGCTTTTGTTATAATTTTTTTTATAGTAATACCTATTATAATTGGGGGGTTTGGAAATTGATTAGTTCCATTAATATTAGGGGCAGGTGATATAGCTTTTCCTCGTATAAATAATTTAAGTTTTTGACTTTTACCACCTTCATTAATATTATTAGTTATATCATCTATGGCTGAGATGGGAGTTGGGGCTGGATGAACAGTTTATCCCCCTCTTGCTTCTTTAGTAGGTCATACGGGAAGAGCAATGGATTTTGCTATTTTTTCATTACATTTAGCTGGGGCTTCTTCTATTATAGGAGCTGTTAATTTTATTACTACTATTATGAATATACGATCTTTTGGAATAATAATGGAAAAGATTTCATTATTTGTTTGGTCTGTTTTAATTACAGCTATTTTATTATTATTATCTTTGCCAGTTTTAGCCGGGGCTATTACTATATTATTAACTGATCGTAATTTTAATACGTCTTTTTTTGACCCTGCTGGGGGAGGTGATCCTATTTTATTTCAACATTTATTTTGATTTTTTGGTCACCC3' (holotype, YHCLU0272; GenBank: OP675437)

5'TCTGGTCAGCTATAGTTGGTACAGCTATAAGAGTTATAATTCGTATAGAATTAGGTCAATCTGGAGCTTTTTTAGGTGATGATCATTTGTATAATGTAGTAGTTACTGCTCATGCTTTTGTTATAATTTTTTTTATAGTAATACCTATTATAATTGGGGGGTTTGGAAATTGATTAGTTCCATTAATATTAGGGGCAGGTGATATAGCTTTTCCTCGTATAAATAATTTAAGTTTTTGACTTTTACCACCTTCATTAATATTATTAGTTATATCATCTATGGCTGAGATGGGAGTTGGGGCTGGATGAACAGTTTATCCCCCTCTTGCTTCTTTAGTAGGTCATACGGGAAGAGCAATGGATTTTGCTATTTTTTCATTACATTTAGCTGGGGCTTCTTCTATTATAGGAGCTGTTAATTTTATTACTACTATTATGAATATACGATCTTTTGGAATAATAATGGAAAAGATTTCATTATTTGTTTGGTCTGTTTTAATTACAGCTATTTTATTATTATTATCTTTGCCAGTTTTAGCCGGGGCTATTACTATATTATTAACTGATCGTAATTTTAATACGTCTTTTTTTGACCCTGCTGGGGGAGGTGATCCTATTTTATTTCAACATTTATTTTGATTTTTTGGTCACCC3' (paratype, YHCLU0273; GenBank: OP675436).

#### Diagnosis

Male of the new species resembles that of *C.caohai* Zhang & Yu, 2020 (Zhang and Yu 2020: 347, figs. 2A–E) in having a blade-shaped RTA and a dagger-shaped embolus, but differs in the following: (1) embolus gradually tapering towards its apex (vs. narrowed in the middle) (cf. Fig. 3D and Zhang and Yu 2020: fig. 2D); (2) conductor nearly triangular, apex sharp and pointing distally (vs. finger-like, apex blunt and pointing prolaterally) (cf. Fig. 3D and E and Zhang and Yu 2020: figs. 2D and E); (3) VTA laminar, relatively large, wider than 1/2 of palpal tibia diameter (vs. papilliform and small, ca. 1/3–1/4 of palpal tibia diameter) (cf. Fig. 3B and Zhang and Yu 2020: fig. 2B). Females of *C.xianning* **sp. nov.** can be easily distinguished from other members of the *C.corticalis*-group, with the exception of *C.altissimus* Hu, 2001 (Hu 2001: 283, fig. 163.1-3) by the atrium represented by two shallow depressions (atrium absent, or present, but represented by one or two deep cavities in all other *corticalis*-group species), differ from *C.altissimus* by: (1) atrium large, nearly as wide as epigynal plate (vs. atrium relatively small, ca. 1/3 of epigyne width) (cf. Fig. 4A and B and Hu 2001: fig. 163.2); (2) spermathecae consisting of head, tubular stalk and base, the two spermathecal heads finger-like, well separated by 1.58 diameters (vs. spermathecae consisting of head and base, the two spermathecal heads reniform, separated by ca. one diameter) (cf. Fig. 4C and D and Hu 2001: fig. 163.3). In addition, *C.xianning* **sp. nov.** also can by separated from *C.caohai* and *C.altissimus* by their habitus: abdomen without distinct colour pattern in *C.xianning* **sp. nov.** (Fig. 4E–H), but with several chevron-shaped bands in *C.caohai* and *C.altissimus* (Zhang and Yu 2020: figs. 2 E–H; Hu 2001: fig. 163.1).

#### Etymology

The specific name refers to the type locality and is a noun in apposition.

#### Distribution

Known from the Mt. Jiugong, Hubei Province, China (Fig. 1).

## Supplementary Material

XML Treatment for Clubiona xianning
